# Network Pharmacology-Based Identification of the Mechanisms of Shen-Qi Compound Formula in Treating Diabetes Mellitus

**DOI:** 10.1155/2020/5798764

**Published:** 2020-06-04

**Authors:** Zhipeng Hu, Maoyi Yang, Liangjun Yang, Chunguang Xie, Hong Gao, Xiaoxu Fu, Hongyan Xie, Ya Liu

**Affiliations:** ^1^Hospital of Chengdu University of Traditional Chinese Medicine, Chengdu 610072, China; ^2^Department of Gastroenterology, Tongde Hospital of Zhejiang Province, Hangzhou 310012, China

## Abstract

**Aim:**

The purpose of this research is to identify the mechanisms of Shen-Qi compound formula (SQC), a traditional Chinese medicine (TCM), for treating diabetes mellitus (DM) using system pharmacology.

**Methods:**

The active components and therapeutic targets were identified, and these targets were analyzed using gene ontology (GO) enrichment analysis, Kyoto Encyclopedia of Genes and Genomes (KEGG) enrichment analysis, and protein-protein interaction (PPI) analysis. Finally, an integrated pathway was constructed to show the mechanisms of SQC.

**Results:**

A total of 282 active components and 195 targets were identified through a database search. The component-target network was constructed, and the key components were screened out according to their degree. Through the GO, PPI, and KEGG analyses, the mechanism network of SQC treating DM was constructed.

**Conclusions:**

This study shows that the mechanisms of SQC treating DM are related to various pathways and targets. This study provides a good foundation and basis for further in-depth verification and clinical application.

## 1. Introduction

Diabetes mellitus (DM) is an endocrine and metabolic disease characterized by persistent hyperglycemia. Its main clinical manifestations include polydipsia, polyphagia, polyuria, and weight change [1]. Due to lifestyle changes, the incidence of DM in China has increased dramatically over the past 30 years. According to epidemiological surveys, China has the largest number of diabetic patients in the world [2, 3]. There are many chronic complications in the later stage of DM, including macrovascular disease, microvascular disease, and diabetic neuropathy [4]. DM and its associated complications have a serious impact on the health of patients and result in a significant economic and health burden on society.

Although the treatment of DM has long been researched, the management of DM and its chronic complications are still big challenges in clinical practice [5–9]. At present, the main objective is to control blood glucose. Therapeutic agents are mainly composed of insulin and oral hypoglycemic medicines. However, these therapeutic drugs have their drawbacks. Insulin is the most effective hypoglycemic agent, but it increases the risk of hypoglycemia, which in turn increases the risk of cardiovascular disease and other complications [10, 11]. However, oral hypoglycemic agents cannot achieve the desired state in many cases, which may be due to their adverse reactions or clinical inertia. Therefore, new means of treatment are needed.

In China, the application of traditional Chinese herbal medicine for DM has a long history. Shen-Qi compound formula (SQC) is a traditional Chinese medicine (TCM) formula that is widely used in treating DM in China. It consists of eight herbal medicines: Radix et Rhizoma Ginseng (RRG, *rén shēn*), Radix Astragali Praeparata cum Melle (RAPM, *huáng qí*), Radix et Rhizoma Salviae Miltiorrhizae (RRSM, *dān shēn*), Radix et Rhizoma Rhei (RRR, *dà huáng*), Rhizoma Dioscoreae (RD, *huái shān yào*), Radix Trichosanthis (RT, *tiān huā fĕn*), Radix Rehmanniae (RR, *dì huáng*), and Fructus Corni (FC, *shān zhū yú*). The composition of SC is shown in Table 1. In a clinical trial involving 219 patients with Type 2 DM, SQC treatment over 12 weeks can significantly reduce fasting and postprandial blood glucose levels as well as significantly improve *β* cell function and insulin resistance [12]. Another clinical trial confirmed the effect of SQC on lowering the blood glucose [13]. Clinical research on the treatment of DM complications with SQC is ongoing (ChiCTR1900026372). Regarding the mechanism, in the above clinical studies, SQC was seen to be able to improve outcomes such as superoxide dismutase (SOD), nitric oxide (NO), reactive oxygen species (ROS), malondialdehyde (MDA), total antioxidant capacity (T-AOC), and glucose like peptide-1. Animal experiments showed that the mechanisms might be related to inhibiting adiponectin, reducing the inflammation and other mechanisms [14, 15]. However, due to the complexity of its components, its comprehensive mechanisms have not yet been fully elucidated.

Network pharmacology comprehensively analyzes many complex networks through a high-throughput analysis and computer calculation. This research idea coincides with the characteristics of the simultaneous effects of multicomponents of TCM. Therefore, it has become an effective tool for the research of traditional Chinese herbal medicine [16].

In this study, we systematically analyzed SQC utilizing network pharmacology to fully elucidate its possible mechanism on DM. Firstly, we screened out the active ingredients of these herbal medicines according to specific criteria. Secondly, the therapeutic targets were obtained. Then, these targets were systematically analyzed, and finally, an integrated pathway was constructed.

## 2. Materials and Methods

### 2.1. Search and Screening of Chemical Constituents

**Figure 1:**
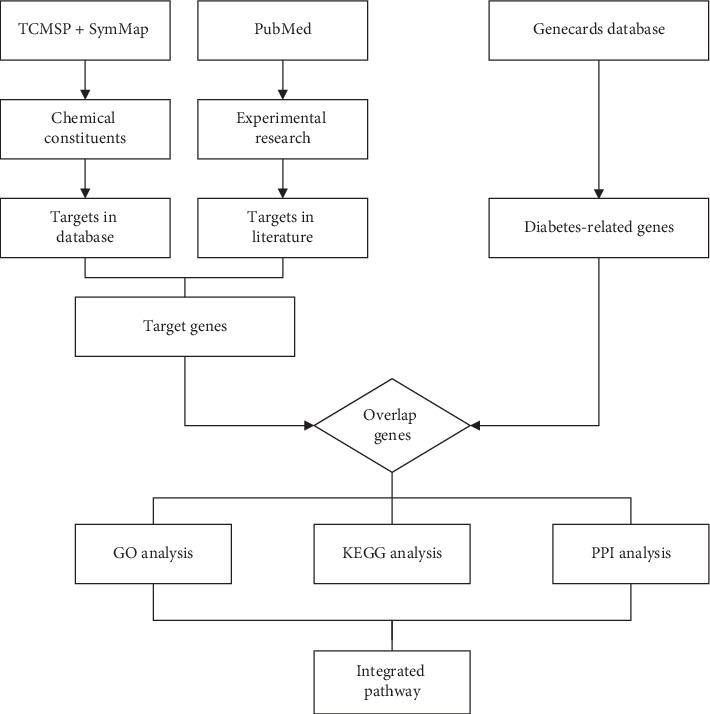
Workflow for the present network pharmacology study.

The workflow for this network pharmacology study can be seen in Figure 1. The screening of the active ingredients of the herbal medicines in SC was conducted on the platform of the TCM System Pharmacology database (TCMSP) (http://lsp.nwu.edu.cn/tcmsp.php) and SymMap (https://www.symmap.org/). TCMSP is a database that is widely used in the network pharmacology research of TCM. It contains information on 499 herbal medicines, 29384 ingredients, and 837 related diseases [17]. This database provides pharmacokinetic information for each compound, which is particularly helpful for us in further screening. SymMap is a database that aims to study the mechanism of TCM from the view point of modern medicine and includes a large amount of information on Chinese herbal medicine, symptoms of TCM, ingredients of medicines, targets, and diseases. In the aspect of drug ingredients, it integrates the information of three databases, TCMID, TCMSP, and TCM-ID, including as many as 19595 ingredients. In terms of disease targets, this database includes information on several commonly used databases, such as HIT, TCMSP, HPO, DrugBank, and NCBI. This database is a useful tool to study the ingredients and targets of TCM [18]. To further screen the active ingredients in the compound, the results retrieved from the databases were screened by two criteria: oral bioavailability (OB) and drug-likeness (DL).

### 2.2. Evaluation of OB

OB represents the proportion of drugs that enter the human blood circulation after oral administration [19]. OB plays an important role in drug delivery. High OB is usually an important characteristic of a chemical component that can be used as a therapeutic drug [20]. OB ≥ 30% was selected as the screening criteria, which is recommended by the TCMSP database [21].

### 2.3. Evaluation of DL

DL is a parameter used to evaluate how “drug-like” a prospective compound is [22]. This concept can help to optimize pharmacokinetics and pharmaceutical properties such as solubility and chemical stability in the drug design [23]. DL was calculated as follows:

(1) $$\begin{array}{c} T\left(A,B\right)=\frac{A\cdot B}{{\left\|A\right\|}^{2}+{\left\|B\right\|}^{2}-A\cdot B}, \end{array}$$

where **A** and **B** represent the molecular property of the herbal ingredients and average molecular property of all the molecules in the DrugBank database (http://www.drugbank.ca/), respectively. DL ≥ 0.18, which was the average DL index of all the compounds in DrugBank, was applied as the screening criteria for the ingredients. To ensure that the results of the study are comprehensive and reliable, some compounds which do not meet the screening criteria but are reported as the main components in the literature have been retained in our research.

### 2.4. Literature Search

As some components with pharmacological effects may not be included in the database or do not meet the conditions of OB and DL, these components will be missed. To avoid this, we searched the medicine of SQC in PubMed for pharmacological research. MeSH terms and free text words were used as the search strategy. Each search term was connected with a Boolean operator to other terms. The time limitation was from January 1, 2000, to February 5, 2020. The full search strategy can be seen in supplementary material 1.

### 2.5. Screening of Targets for Ingredients

We searched the TCMSP database for protein targets of the chemical constituents we collected. Then, the UniProt Knowledgebase (https://www.uniprot.org/) was used to search the corresponding human gene names. UniProt Knowledgebase is a protein database with extensive amount of information and resources. It integrates data from three databases: Swiss-Prot, TrEMBL, and PIR-PSD. Its data mainly come from the protein sequence obtained after the completion of the genome sequencing project. It contains a large amount of information about the biological functions of proteins from the literature. We retrieved information about proteins and replaced the protein targets with corresponding gene targets in this database.

### 2.6. Screening of Targets for DM

We then searched about the targets for DM. These were retrieved from the Human Gene Database (Genecards database, https://www.genecards.org/). GeneCards is a searchable, integrative database that provides comprehensive, user-friendly information on all annotated and predicted human genes. It integrates gene-centric data from about 150 databases, including NCBI, UCSC, and Nsembl [24–26]. Diabetes-related target genes were retrieved using the keywords “diabetes” or “diabetes mellitus”. The score ranks diseases by how closely they are associated with the gene, factoring in the relative reliability of the sources, and a criteria score >5. This is widely used in network pharmacology research and applied to further screen the target genes [27, 28].

### 2.7. Protein-Protein Interaction Network Construction

The protein-protein interaction (PPI) network analysis helps study the molecular mechanism of diseases and discover new drug targets from a systematic perspective. We constructed a PPI network for the protein targets of SQC, which we retrieved in the TCMSP database. This was done using the string database (https://string-db.org/). This database is used for searching and predicting protein interactions. It can be applied to 2031 species and includes 9.6 million protein interactions and 13.8 million protein interactions. In addition to the experimental data, the results of text mining from PubMed abstracts, and the synthesis of other database data, it also uses bioinformatic methods to predict the results. This is one of the most diverse and informative websites for PPI analysis. The line thickness indicates the strength of data support, that is, confidence. The minimum required interaction score is medium confidence (0.400), which is also the default threshold.

### 2.8. Gene Ontology Enrichment and Kyoto Encyclopedia of Gene and Genome Pathway Enrichment

To elucidate the mechanism of SQC in treating DM, we collected the intersected target genes for gene ontology (GO) enrichment and Kyoto Encyclopedia of Gene and Genome (KEGG) pathway enrichment analysis. GO enrichment is the description of genes in different dimensions and levels such as the cellular components (CC), biological process (BP), and molecular function (MF). Through the GO analysis, we can get a better understanding of genes [29]. KEGG analysis refers to the analysis of the pathways involved in genes. Through the KEGG analysis, we can fully understand the relevant pathways in biological processes. GO enrichment and KEGG pathway enrichment analyses were performed on the Database for Annotation, Visualization, and Integrated Discovery (DAVID 6.8, https://david.ncifcrf.gov/). DAVID is a robust, publicly available database that aims to illuminate how environmental chemicals affect human health [30, 31]. In the results of the GO and KEGG analyses, the *P* value indicates whether the pathway is significantly related to SQC, and the count value reflects the number of targets on the pathway. The larger the count value is, the more targets SQC has on the pathway. We filtered according to a *P* value of <0.01 and arranged the filtered results according to the count. For the GO analysis, we selected the top five results for each item to display. For the KEGG analysis, to provide readers with a more comprehensive understanding of the mechanism of SQC, the top 25 entries in the KEGG pathways were exhibited.

### 2.9. Network Construction

To display the above results more vividly, compound-target (C-T) network and target-pathway (T-P) network diagrams for the above results were constructed. These network diagrams were drawn using Cytoscape version 3.6.1, which can graphically display the network and edit it [32]. In these graphs, compounds and targets are represented by nodes and their interactions are reflected by edges.

### 2.10. Signal Pathway Construction of SQC in Treating DM

We excluded the pathways that were unrelated to diabetes among the top 25 pathways. The rest of the pathways were considered to be the main mechanisms. Then, we looked up the KEGG database to understand the specific processes of these mechanisms. Due to the complexity of mechanisms, only the most commonly used and representative molecules in pathways and proven interactions were selected in the construction of the signal pathway. Special attention was paid to the interaction between signal pathways. In this way, we integrated the rest of the diabetic signal pathways and constructed the pathway network of SQC in the treatment of DM.

## 3. Results

### 3.1. Active Ingredients in SQC

After a preliminary search, a total of 959 chemical ingredients were found in the TCMSP database and 1183 ingredients in SymMap. The information of ingredients retrieved in TCMSP and SymMap can be viewed in the supplementary materials 2 and 3, respectively. Then, the retrieval results of the two databases are combined. A total of 516 ingredients remained including 70 ingredients in *rén shēn*, 19 in *dì huáng*, 103 in *shān zhū yú*, 175 in *dān shēn*, 52 in *huáng qí*, 9 in *tiān huā fĕn*, 154 in *huái shān yào*, and 19 in *dahuang*. After a literature search, 30 additional ingredients were added to our study including 10 in RRG (Ginsenoside Re [33], ginseng total saponin [34], Ginsenoside-Rg3 [35–37], ginsenoside Rh2 [38, 39], ginsenoside Rb2 [40], Ginsenoside Rb1 [41–43], Compound K [44], Ginsenoside Rg1 [45, 46], Ginseng polysaccharides [47, 48], and Malonylginsenoside Rc), 3 in RAPM (Astragalus polysaccharide [49–66], Astragaloside Iv [67–72], and astragalin [73]), 4 in RRSM (danshensu [74], Lithospermic acid B [75, 76], Magnesium Lithospermate B [77, 78], and salvianolic acid B [79–85]), 3 in RRR (rhein [86], emodin [87, 88], and Argirein [89]), 2 in RD (Allantoin [90, 91], Dioscorea opposita Polysaccharide [92–94]), 1 in RR (catalpol [95–103]), 7 in FC (5-hydroxymethylfurfural [104], Iridoid glycoside [105], Loganin [106], 7-O-Galloyl-D-sedoheptulose [107, 108], total triterpene acids [109], iridoid total glycoside [110], and cornuside). After removing the duplicates, a total of 352 nonrepeating ingredients were retrieved.

### 3.2. Target Retrieval and Analysis

**Figure 2:**
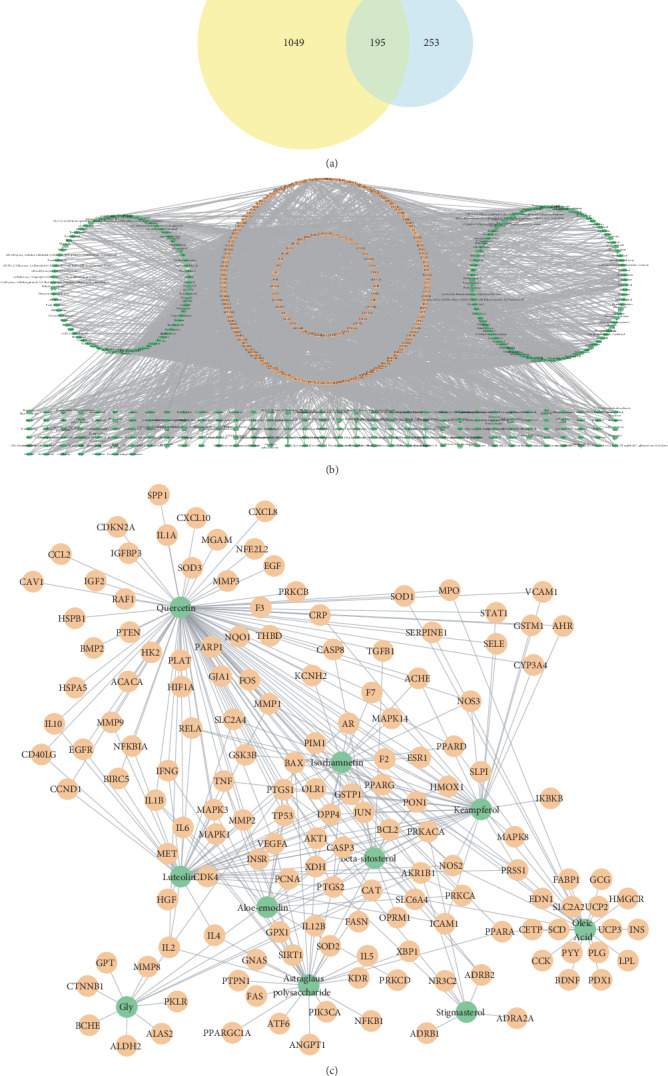
Network construction. (a) Venn diagram of diabetes-related targets and SQC-related targets. The yellow circle represents diabetes-related targets, and the blue circle represents SQC-related targets. (b) The network of compound-target in SQC. The green nodes represent the active compound in SQC. The blue nodes represent the potential targets. The edges represent the interaction between the blue and green nodes. (c) Top 10 ingredients and their targets. The green nodes represent the ingredients, the orange nodes represent the targets, and the edges represent the interaction between them.

Based on the above results, we further searched the targets of these ingredients. A total of 5201 targets were retrieved. Four-hundred and forty-eight targets remained after eliminating the duplicate targets. Then, DM-related targets were searched in the platform, Genecards. A total of 1244 targets were retrieved in the database using the keyword “diabetes”. By intersecting the target of SQC with that of DM, a total of 195 overlapping target genes were screened out. The targets of SQC and DM can be viewed in supplementary material 4. These targets are considered to be the active targets of SQC in the treatment of DM, as shown in Figure 2(a). Then, we further compared the active target we obtained with the original targets of SQC. Components that do not have active targets will be considered as ineffective ingredients. Eventually, 282 ingredients were retained, which were considered to be active ingredients in the treatment of DM by SQC. These results could help us understand the mechanisms likely to be involved in its therapeutic effect. A network of active compound-target was constructed according to these results (Figure 2(b)). Then, we ranked these ingredients according to the degree. The top 10 ingredients included kaempferol, supervisolin, Gly, beta-sitosterol, stigmasterol, astragalus polysaccharide, oleic acid, isorhamnetin, and aloe-emodin.  Figure 2(c) shows the interaction between these ingredients and their targets.

### 3.3. GO Enrichment and KEGG Analysis

**Figure 3:**
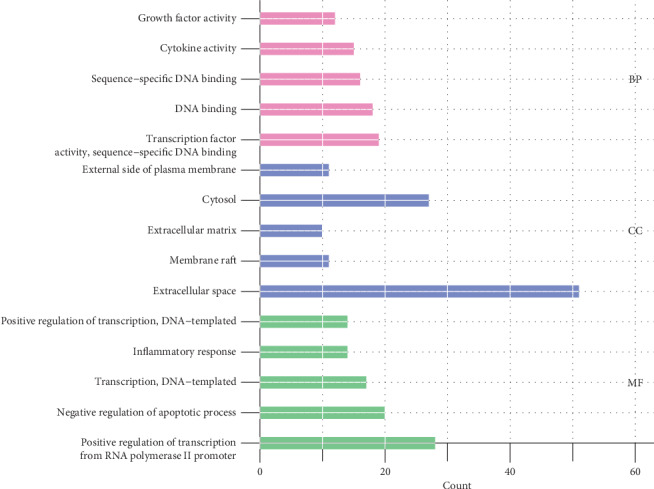
GO enrichment analysis of the SQC-related target genes in the treatment of DM. The *X*-axis represents the gene count of each GO term, and the *Y*-axis represents the categories in the cellular component, molecular function, and biological process. All the categories were screened using the criteria of a *P* value < 0.01.

To further elucidate the molecular mechanism(s) of SQC in the treatment of DM, GO enrichment and KEGG pathway analyses were performed on the selected candidate targets in the CTD database. The GO analysis describes the function of candidate targets from three aspects: biological process (BP), cellular component (CC), and molecular function (MF). A *P* value of <0.01 was considered statistically significant. The complete results of the GO enrichment analysis are displayed in supplementary materials 5. The top five results for the three aspects are shown in Figure 3. According to the results of the GO enrichment analysis, the biological processes involved are mainly the positive regulation of transcription from RNA polymerase II promoter (GO: 0045944), negative regulation of apoptotic process (GO: 0043066), transcription, DNA-templated (GO: 0006351), inflammatory response (GO: 0006954), positive regulation of transcription, and DNA-templated (GO: 0045893). The enriched molecular functional ontologies mainly include transcription factor activity, sequence-specific DNA binding (GO: 0003700), DNA binding (GO: 0003677), sequence-specific DNA binding (GO: 0043565), cytokine activity (GO: 0005125), and growth factor activity (GO: 0008083). The cellular component analysis showed that extracellular space (GO: 0005615), membrane raft (GO: 0045121), extracellular matrix (GO: 0031012), cytosol (GO: 0005829), and external side of the plasma membrane (GO: 0009897) were the most important.

**Figure 4:**
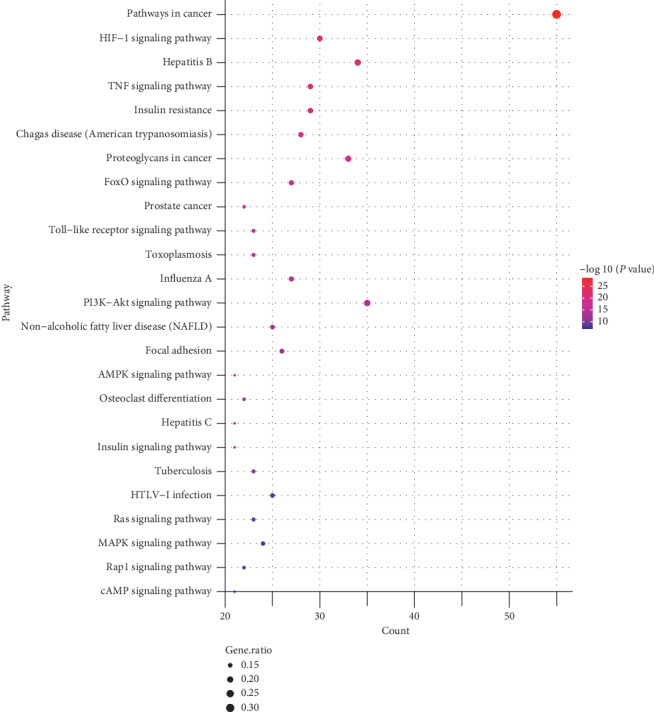
Bubble chart of the KEGG enrichment analysis of the SQC-related target genes in the treatment of DM. The *X*-axis represents the count of genes of each GO term, the *Y*-axis represents the categories in the KEGG analysis, the size of the nodes represents the gene ratio, and the color of the nodes was determined by the value of −log10 (*P* value).

Subsequently, we analyzed the KEGG pathway of the candidate genes. The results of the KEGG analysis are displayed in supplementary material 6. We sequenced these pathway analysis results by the count of genes.  Figure 4 shows the 25 most prominent signal pathways. According to these results, the most important pathways related to treating of diabetes by SQC are PI3K-Akt, HIF-1, TNF, insulin resistance, FoxO, MAPK, AMPK, and insulin signal pathways.

**Figure 5:**
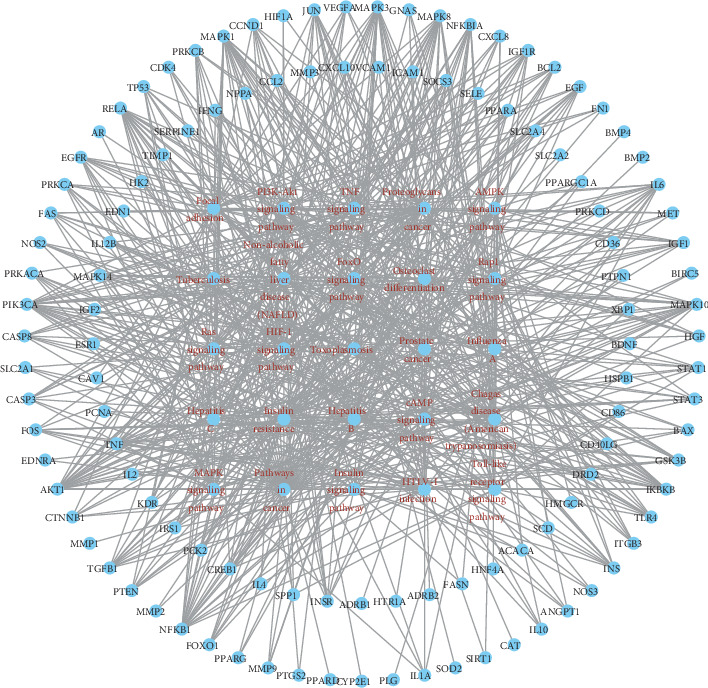
The network of the target-pathway in SQC. The red nodes represent the pathway of SQC in the treatment of DM. The blue nodes represent the target genes. The edges represent the interaction between the red and blue nodes.

To show the relationship between these 25 pathways and their related genes, we have mapped the T-P network (Figure 5). In this, the two outer circles represent the genes associated with pathways. The innermost circle represents the most prominent 25 signal pathways.

### 3.4. PPI Network for the Targets of SQC

**Figure 6:**
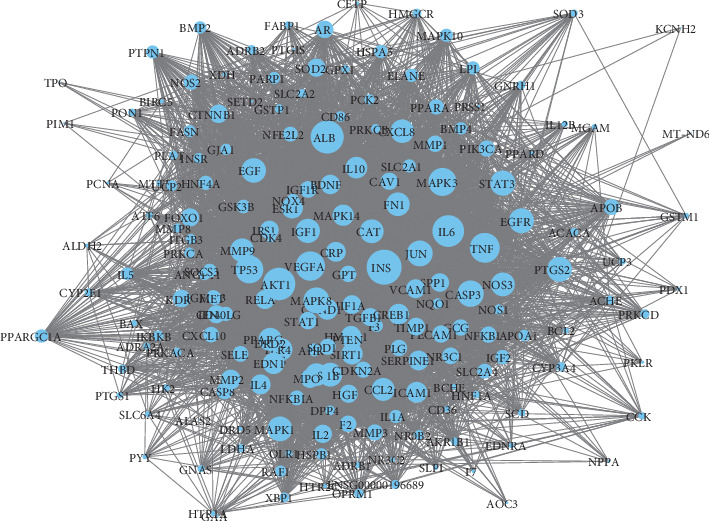
PPI network of the target genes. The size of the nodes is proportional to their degrees.

**Figure 7:**
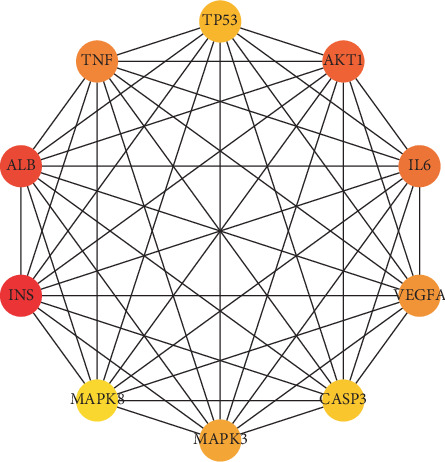
Top 10 hub genes in the PPI network.

Then, we conducted a PPI network analysis. As is shown in Figure 6, there are 164 nodes, 3684 edges, and an average node of 44.9. The top ten genes according to the degree are as follows: AKT1, interleukin-6 (IL6), albumin (ALB), insulin (INS), vascular endothelial growth factor *α* (VEGFA), tumor necrosis factor (TNF), tumor protein P53 (TP53), caspase 3 (CASP3), mitogen-activated protein kinase 3 (MAPK3), and mitogen-activated protein kinase 8 (MAPK8). These genes are considered to play an important role in treating of DM (Figure 7).

### 3.5. Construction of an Integrated Pathway for SQC

**Figure 8:**
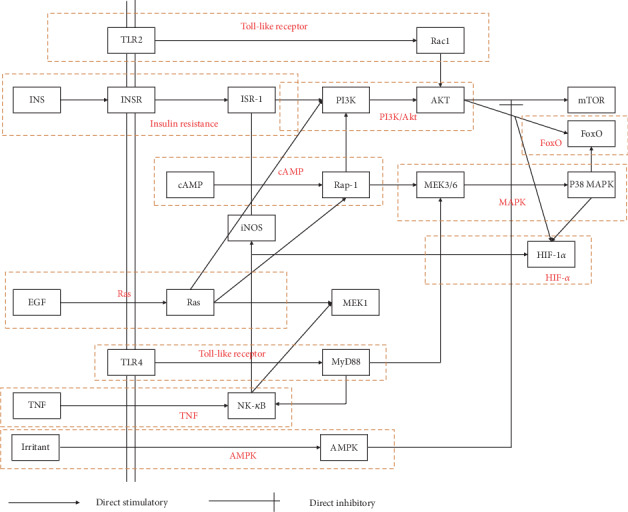
The integrated pathway and potential mechanism(s) of SQC in the treatment of DM.

According to the KEGG results, an integrated pathway was constructed by combining the key pathways in the treatment of DM, including PI3k-Akt, TNF, HIF-α, insulin resistance, FoxO, toll-like Ras, Rap-1, AMPK, MAPK, and cAMP signal pathways (Figure 8).

## 4. Discussion

In this study, the mechanism of SQC in the treatment of DM was comprehensively identified using network pharmacology. Some interesting results were found in our research, which will be helpful in our further investigations.

A total of 352 nonrepeating ingredients including 282 active ingredients were identified through database and literature search. The components of TCM formula include not only the compounds contained in each herbal medicine but also the new components generated in the preparation of the formula. In this research, new ingredients generated during the preparation process were not investigated. In future research, more attention should be paid to these newly generated compounds.

There were 150 targets in all of the top 10 ingredients, and all of these 10 ingredients have multiple targets. The analysis of these targets shows that these mainly exert antidiabetic effects through various pathways, such as the TNF, PI3K/Akt, insulin resistance, and HF-1 signal pathways. By directly affecting several key proteins in these pathways, such as Akt, TNF, and MAPK, these ingredients can directly affect the activity of the abovementioned multiple diabetes-related signal pathways. Multiple ingredients act on the same target, indicating that these components have synergistic effects in treating diabetes.

The GO analysis describes the target of SQC from the aspects of the biological process, molecular function, and cell components. In terms of cell components, the related targets of SQC are located in the extracellular space. If the product of a gene is secreted from cells to tissue fluid or blood, then generally, the gene will be annotated as extracellular space. In the physiological process of diabetes, insulin is secreted from the cell after synthesis. Also, diabetes is an inflammatory disease. Many inflammatory cytokines are secreted into the blood from the cell. These targets were classified as extracellular space. The results of the GO analysis suggest that SQC may have a regulatory effect on these processes.

By analyzing the molecular function of the target gene, we can further understand the biological process of its main ingredients. The main molecular function of SQC is to regulate RNA polymerase, affect apoptosis, and reduce inflammation. This result is consistent with the biological process. The transcription process of an organism is mainly initiated by the formation of transcription initiation complex by transcription factors and RNA polymerase II, which are the bound to the downstream DNA. In DM, the FoxO transcription factor is a key molecule in the insulin or insulin-like growth factor signal pathway, and the p65 transcription factor is an important transcription factor that helps to regulate inflammation. The PI3K/Akt signal pathway can affect many downstream transcription factors and biological processes, including apoptosis and inflammation. Chronic inflammation and apoptosis of *β* cells will have a significant impact on the insulin signal of cells and eventually lead to diabetes. The results of the GO analysis indicated that SQC might have an impact on biological processes like *β* cell apoptosis chronic inflammation.

The KEGG analysis provided a further understanding of the mechanism of SQC based on the GO analysis. The PI3K/Akt signal pathway is one of the most important signal pathways in the pathogenesis of diabetes. It is a significant pathway in cell mobilization, migration, differentiation, and antiapoptosis. It also plays an important role in the regulation of glucose transport, glycogen synthesis, glycolysis, and gluconeogenesis, as well as in the process of protein synthesis and fat decomposition. The KEGG analysis showed that SQC has 35 PI3K/Akt-related targets. This indicates that SQC can regulate the apoptosis and metabolism of diabetes.

Chronic inflammation can affect the insulin sensitivity of the body and even lead to the occurrence of insulin resistance. TNF is one of the most important signal pathways in inflammation. It can not only activate the downstream inflammatory response, leading to the aggravation and chronicity of inflammation but also directly affect the insulin signal pathway and aggravate insulin resistance. Furthermore, the TNF pathway can directly affect the MAPK signal pathway, which is closely related to cell proliferation and apoptosis. Through these mechanisms, the activity of the TNF signal pathway has a significant impact on DM. SQC can improve the insulin resistance of DM by affecting the TNF signal pathway, which is consistent with the previous clinical research results.

Hypoxia-inducible factor-1 *α* (HIF-1 *α*) is an important transcription factor, which can regulate the expression of many downstream target genes and participate in hypoxia adaptation, angiogenesis, immune response, apoptosis, and other reactions. It plays an important role in diabetes. The changes of HIF-1 *α* expression level in DM, together with the structural and functional abnormalities caused by the covalent modification of glyoxal, ultimately lead to disorder of the signal pathway regulation, such as angiogenesis and apoptosis. These are important molecular events in the occurrence and progress of diabetes and its complications. It was found that the TNF, PI3K/Akt, and MAPK signal pathways can interact with the HIF signal pathway, leading to the gradual progress of the disease.

Among the signal pathways we obtained through the KEGG analysis, the toll-like receptor and cAMP and Ras pathways are important signal pathways for organisms, which can participate in a variety of biological processes and have mutual relationships with multiple signal pathways. For example, toll-like receptor-mediated pattern recognition can rapidly activate the immune response, NF-*κ*B signal pathway, and MAPK signal pathway downstream of TNF, thus leading to inflammation and apoptosis.

The PPI analysis further explored the interaction of these targets. From these results, we can know which targets play a more important role. TNF, Akt, MAPK, and other targets are the most important proteins. This result is consistent with the results of the KEGG analysis.

## 5. Conclusions

This research systematically investigated the ingredients and targets of SQC in the treatment of DM through the combination of network pharmacology and a literature search. A total of 282 components and 195 targets were obtained. The multitarget mechanisms of SQC were studied using the GO, KEGG, and PPI analyses. This study provides a solid foundation for further experimental verification and clinical application. Further experimental verification is needed to confirm the result of this current study.

## Figures and Tables

**Table 1:** The composition of SC.

Pharmaceutical	Pīn yīn	Composition (%)
Radix et Rhizoma Ginseng	*rén shēn*	17.4
Radix Astragali Praeparata cum Melle	*huáng qí*	17.4
Radix et Rhizoma Salviae Miltiorrhizae	*dān shēn*	11.6
Radix et Rhizoma Rhei	*dà huáng*	2
Rhizoma Dioscoreae	*huái shān yào*	11.6
Radix Trichosanthis	*tiān huā fĕn*	11.6
Radix Rehmanniae Recens	*dì huáng*	11.6
Fructus Corni	*shān zhū yú*	6.9

## Data Availability

The complete information of active ingredients, MOL_ID, molecule names, targets, and gene code, and GO and KEGG analysis results can be seen in supplementary materials.

## Supplementary Materials

Supplementary Materials Supplementary material 1: the search strategies for eight herbal medicines in SQC. Supplementary material 2: the information of ingredients retrieved in TCMSP. Supplementary material 3: the information of ingredients retrieved in SymMap. Supplementary material 4: summarized target information, including the targets of SQC and diabetes. Supplementary material 5: raw data of GO enrichment analysis. Supplementary material 6: raw data of KEGG enrichment analysis.
