# Supplementary material for: Network Pharmacology-Based Identification of the Mechanisms of Shen-Qi Compound Formula in Treating Diabetes Mellitus
Source: Evid Based Complement Alternat Med. 2020 Jun 4;2020:5798764. doi: 10.1155/2020/5798764 (PMC7292981; doi:10.1155/2020/5798764)
Supplement: Supplementary Materials — Supplementary material 1: the search strategies for eight herbal medicines in SQC. Supplementary material 2: the information of ingredients retrieved in TCMSP. Supplementary material 3: the information of ingredients retrieved in SymMap. Supplementary material 4: summarized target information, including the targets of SQC and diabetes. Supplementary material 5: raw data of GO enrichment analysis. Supplementary material 6: raw data of KEGG enrichment analysis. [file 5798764.f1.zip › 5798764.f1/Supplementary material 1.docx]

**Search strategy of *rén shēn***

(((((((((((((((((((("Panax"[Mesh]) OR Panaxs[Title/Abstract]) OR Ninjin[Title/Abstract]) OR Ninjins[Title/Abstract]) OR Schinseng[Title/Abstract]) OR Schinsengs[Title/Abstract]) OR Renshen[Title/Abstract]) OR Renshens[Title/Abstract]) OR Jen Shen[Title/Abstract]) OR Jen Shens[Title/Abstract]) OR Shen, Jen[Title/Abstract]) OR Shens, Jen[Title/Abstract]) OR Ginseng[Title/Abstract]) OR Ginsengs[Title/Abstract]) OR Shinseng[Title/Abstract]) OR Shinsengs[Title/Abstract]) OR Panax ginseng[Title/Abstract]) OR Panax ginsengs[Title/Abstract]) OR ginsengs, Panax[Title/Abstract])) AND diabetes mellitus[MeSH Terms]

**Search strategy of *huáng qí***

(((((((((((((((("Huang Qi"[Supplementary Concept] OR (Astragali[All Fields] AND ("plant roots"[MeSH Terms] OR ("plant"[All Fields] AND "roots"[All Fields]) OR "plant roots"[All Fields] OR "radix"[All Fields])) AND ("astragalus propinquus"[MeSH Terms] OR ("astragalus"[All Fields] AND "propinquus"[All Fields]) OR "astragalus propinquus"[All Fields] OR ("astragalus"[All Fields] AND "membranaceus"[All Fields]) OR "astragalus membranaceus"[All Fields]) AND Title/Abstract[All Fields]) OR Astragalus membranaceus root[Title/Abstract]) OR (Phaca[All Fields] AND membranacea[All Fields] AND root[Title/Abstract])) OR ((Mo, Jia[Full Author Name] OR Jia, Mo[Full Author Name]) AND Huang Qi[Title/Abstract])) OR ("Huang Qi"[Supplementary Concept] OR "Huang Qi"[All Fields] OR "huang qi"[All Fields]) AND ("astragalus propinquus"[MeSH Terms] OR ("astragalus"[All Fields] AND "propinquus"[All Fields]) OR "astragalus propinquus"[All Fields]) AND Title/Abstract[All Fields]) OR (("talus"[MeSH Terms] OR "talus"[All Fields] OR "astragalus"[All Fields] OR "astragalus plant"[MeSH Terms] OR ("astragalus"[All Fields] AND "plant"[All Fields]) OR "astragalus plant"[All Fields]) AND ("plant roots"[MeSH Terms] OR ("plant"[All Fields] AND "roots"[All Fields]) OR "plant roots"[All Fields] OR "root"[All Fields])) AND ("astragalus propinquus"[MeSH Terms] OR ("astragalus"[All Fields] AND "propinquus"[All Fields]) OR "astragalus propinquus"[All Fields]) AND Title/Abstract[All Fields]) OR Astragalus membranaceus root powder[Title/Abstract]) OR Astragalus membranaceus root extract[Title/Abstract]) OR ("Huang Qi"[Supplementary Concept] OR "Huang Qi"[All Fields] OR "huang qi"[All Fields]) AND (("astragalus propinquus"[MeSH Terms] OR ("astragalus"[All Fields] AND "propinquus"[All Fields]) OR "astragalus propinquus"[All Fields] OR ("astragalus"[All Fields] AND "mongholicus"[All Fields]) OR "astragalus mongholicus"[All Fields]) AND Bunge[All Fields]) AND Title/Abstract[All Fields]) OR (RAH[All Fields] AND extract[Title/Abstract])) OR Radix Astragali seu Hedysari[Title/Abstract]) OR Radix Astragali[Title/Abstract]) OR membranous milkvetch root[Title/Abstract]) OR Astragalus propinquus root[Title/Abstract]) OR Astragalus[Title/Abstract]) OR Astragalan[Title/Abstract]) AND "diabetes mellitus"[MeSH Terms]

**Search strategy of *dì huáng***

((((Rehmannia[Title/Abstract] OR (glutinous[All Fields] AND rehmannia[Title/Abstract])) OR Radix Rehmanniae Preparata[Title/Abstract]) OR Di Huang[Title/Abstract]) OR dihuang[Title/Abstract]) AND "diabetes mellitus"[MeSH Terms]

**Search strategy of *shān zhū yú***

(((shan zhuyu[Title/Abstract]) OR Cornus officinalis[Title/Abstract])) AND diabetes mellitus[MeSH Terms]

**Search strategy of *dān shēn***

(((((((((("Salvia miltiorrhiza"[Mesh] OR (Tan[All Fields] AND Seng[Title/Abstract])) OR Dan-Shen[Title/Abstract]) OR Dan Shen[Title/Abstract]) OR Chinese Salvia[Title/Abstract]) OR (("asian continental ancestry group"[MeSH Terms] OR ("asian"[All Fields] AND "continental"[All Fields] AND "ancestry"[All Fields] AND "group"[All Fields]) OR "asian continental ancestry group"[All Fields] OR "chinese"[All Fields]) AND Salvias[Title/Abstract])) OR (("salvia"[MeSH Terms] OR "salvia"[All Fields]) AND Chinese[Title/Abstract])) OR (("salvia"[MeSH Terms] OR "salvia"[All Fields] OR "salvias"[All Fields]) AND Chinese[Title/Abstract])) OR Salvia miltiorrhizae[Title/Abstract]) OR Danshen[Title/Abstract]) OR salvia[Title/Abstract]) AND "diabetes mellitus"[MeSH Terms]

**Search strategy of *tiān huā fĕn***

(((((((("radix Trichosanthis"[Supplementary Concept] OR Trichosanthin[Title/Abstract]) OR alpha-Trichosanthin[Title/Abstract]) OR alpha Trichosanthin[Title/Abstract]) OR Compound Q[Title/Abstract]) OR Tian-hua-fen[Title/Abstract]) OR GLQ-223[Title/Abstract]) OR GLQ 223[Title/Abstract]) OR GLQ223[Title/Abstract]) AND "diabetes mellitus"[MeSH Terms]

**Search strategy of *dà huáng***

((((((((("Rheum"[Mesh] OR Rhubarb[Title/Abstract]) OR Rheum officinale[Title/Abstract]) OR Da Huang[Title/Abstract]) OR (Huang[All Fields] AND Da[Title/Abstract])) OR Chinese Rhubarb[Title/Abstract]) OR (("rheum"[MeSH Terms] OR "rheum"[All Fields] OR "rhubarb"[All Fields]) AND Chinese[Title/Abstract])) OR Rheum rhaponticum[Title/Abstract]) OR Rheum tanguticum[Title/Abstract]) OR Radix et Rhizoma Rhei[Title/Abstract]) AND "diabetes mellitus"[MeSH Terms]

**Search strategy of *huái shān yào***

((((((((((((((((((("Dioscorea"[Mesh] OR Dioscorea villosa[Title/Abstract]) OR Wild Yam[Title/Abstract]) OR Wild Yams[Title/Abstract]) OR (Yam[All Fields] AND Wild[Title/Abstract])) OR (("dioscorea"[MeSH Terms] OR "dioscorea"[All Fields] OR "yams"[All Fields]) AND Wild[Title/Abstract])) OR Dioscorea opposita[Title/Abstract]) OR Dioscorea polystachya[Title/Abstract]) OR Chinese Yam[Title/Abstract]) OR Chinese Yams[Title/Abstract]) OR (Yam[All Fields] AND Chinese[Title/Abstract])) OR (("dioscorea"[MeSH Terms] OR "dioscorea"[All Fields] OR "yams"[All Fields]) AND Chinese[Title/Abstract])) OR Shan Yao[Title/Abstract]) OR (Shan[All Fields] AND Yaos[Title/Abstract])) OR (Yao[All Fields] AND Shan[Title/Abstract])) OR (Yaos[All Fields] AND Shan[Title/Abstract])) OR Dioscorea batatas[Title/Abstract]) OR Yam[Title/Abstract]) OR Yams[Title/Abstract]) OR Dioscoreae[Title/Abstract]) AND "diabetes mellitus"[MeSH Terms]
