# Supplementary material for: Network Pharmacology-Based Identification of the Mechanisms of Shen-Qi Compound Formula in Treating Diabetes Mellitus
Source: Evid Based Complement Alternat Med. 2020 Jun 4;2020:5798764. doi: 10.1155/2020/5798764 (PMC7292981; doi:10.1155/2020/5798764)
Supplement: Supplementary Materials — Supplementary material 1: the search strategies for eight herbal medicines in SQC. Supplementary material 2: the information of ingredients retrieved in TCMSP. Supplementary material 3: the information of ingredients retrieved in SymMap. Supplementary material 4: summarized target information, including the targets of SQC and diabetes. Supplementary material 5: raw data of GO enrichment analysis. Supplementary material 6: raw data of KEGG enrichment analysis. [file 5798764.f1.zip › 5798764.f1/Supplementary material 2.docx]

| Mol ID | Molecule Name | MW | AlogP | Hdon | Hacc | OB (%) | Caco-2 | BBB | DL | FASA- | HL | Herb |
| --- | --- | --- | --- | --- | --- | --- | --- | --- | --- | --- | --- | --- |
| MOL000358 | beta-sitosterol | 414.79 | 8.08 | 1 | 1 | 36.91 | 1.32 | 0.99 | 0.75 | 0.23 | 5.36 | RRR、RRG、FC |
| MOL001771 | poriferast-5-en-3beta-ol | 414.79 | 8.08 | 1 | 1 | 36.91 | 1.45 | 1.14 | 0.75 | 0 | 5.07 | FC、RRSM |
| MOL002879 | Diop | 390.62 | 7.44 | 0 | 4 | 43.59 | 0.79 | 0.26 | 0.39 | 0.28 | 3.6 | FC、RRG |
| MOL000359 | sitosterol | 414.79 | 8.08 | 1 | 1 | 36.91 | 1.32 | 0.87 | 0.75 | 0.22 | 5.37 | FC、RR |
| MOL005360 | malkangunin | 432.56 | 1.84 | 2 | 7 | 57.71 | 0.22 | -0.17 | 0.63 | 0.3 | 4.09 | FC、RRG |
| MOL000554 | gallic acid-3-O-(6'-O-galloyl)-glucoside | 484.4 | -0.03 | 9 | 14 | 30.25 | -1.96 | -2.76 | 0.67 | 0.36 | 2.48 | FC、RRR |
| MOL000449 | Stigmasterol | 412.77 | 7.64 | 1 | 1 | 43.83 | 1.44 | 1 | 0.76 | 0.22 | 5.57 | RR、RRG、RD、FC |
| MOL002235 | EUPATIN | 360.34 | 1.99 | 3 | 8 | 50.8 | 0.53 | -0.26 | 0.41 | 0.22 | 13.94 | RRR |
| MOL002251 | Mutatochrome | 552.96 | 10.9 | 0 | 1 | 48.64 | 1.97 | 0.84 | 0.61 | 0.32 | 15.73 | RRR |
| MOL002259 | Physciondiglucoside | 608.6 | -0.91 | 8 | 15 | 41.65 | -2.64 | -3.43 | 0.63 | 0.3 | 27.61 | RRR |
| MOL002260 | Procyanidin B-5,3'-O-gallate | 730.67 | 4.6 | 12 | 16 | 31.99 | -1.61 | -2.88 | 0.32 | 0.39 | 5.98 | RRR |
| MOL002268 | rhein | 284.23 | 1.88 | 3 | 6 | 47.07 | -0.2 | -0.99 | 0.28 | 0.47 | 32.12 | RRR |
| MOL002276 | Sennoside E_qt | 524.5 | 3.91 | 6 | 9 | 50.69 | -0.74 | -1.56 | 0.61 | 0.37 | 33.6 | RRR |
| MOL002280 | Torachrysone-8-O-beta-D-(6'-oxayl)-glucoside | 480.46 | 0.64 | 5 | 12 | 43.02 | -1.23 | -1.84 | 0.74 | 0.32 | 16.29 | RRR |
| MOL002281 | Toralactone | 272.27 | 2.25 | 2 | 5 | 46.46 | 0.86 | 0.37 | 0.24 | 0.35 | 3.55 | RRR |
| MOL002288 | Emodin-1-O-beta-D-glucopyranoside | 432.41 | 0.59 | 6 | 10 | 44.81 | -1.12 | -2 | 0.8 | 0.34 | 29.79 | RRR |
| MOL002293 | Sennoside D_qt | 524.5 | 3.91 | 6 | 9 | 61.06 | -0.7 | -1.46 | 0.61 | 0.39 | 33.92 | RRR |
| MOL002297 | Daucosterol_qt | 386.73 | 7.67 | 1 | 1 | 35.89 | 1.35 | 1.07 | 0.7 | 0.2 | 6.12 | RRR |
| MOL002303 | palmidin A | 510.52 | 4.52 | 6 | 8 | 32.45 | -0.36 | -1.47 | 0.65 | 0.4 | 32.14 | RRR |
| MOL000471 | aloe-emodin | 270.25 | 1.67 | 3 | 5 | 83.38 | -0.12 | -1.07 | 0.24 | 0 | 31.49 | RRR |
| MOL000096 | (-)-catechin | 290.29 | 1.92 | 5 | 6 | 49.68 | -0.03 | -0.78 | 0.24 | 0.35 | 0.38 | RRR |
| MOL000472 | emodin | 270.25 | 2.49 | 3 | 5 | 24.4 | 0.22 | -0.66 | 0.24 | 0 |  | RRR |
| MOL000006 | luteolin | 286.25 | 2.07 | 4 | 6 | 36.16 | 0.19 | -0.84 | 0.25 | 0.39 | 15.94 | RRSM |
| MOL000569 | digallate | 322.24 | 1.53 | 6 | 9 | 61.85 | -0.76 | -1.52 | 0.26 | 0.43 | 5.29 | RRSM |
| MOL001601 | 1,2,5,6-tetrahydrotanshinone | 280.34 | 2.98 | 0 | 3 | 38.75 | 0.96 | 0.39 | 0.36 | 0.33 | 18.05 | RRSM |
| MOL001659 | Poriferasterol | 412.77 | 7.64 | 1 | 1 | 43.83 | 1.44 | 1.03 | 0.76 | 0.22 | 5.34 | RRSM |
| MOL001942 | isoimperatorin | 270.3 | 3.65 | 0 | 4 | 45.46 | 0.97 | 0.66 | 0.23 | 0.27 | -1.44 | RRSM |
| MOL002222 | sugiol | 300.48 | 4.99 | 1 | 2 | 36.11 | 1.14 | 0.7 | 0.28 | 0.27 | 14.62 | RRSM |
| MOL002651 | Dehydrotanshinone II A | 292.35 | 4.22 | 0 | 3 | 43.76 | 1.02 | 0.52 | 0.4 | 0.33 | 23.71 | RRSM |
| MOL002776 | Baicalin | 446.39 | 0.64 | 6 | 11 | 40.12 | -0.85 | -1.74 | 0.75 | 0.36 | 17.36 | RRSM |
| MOL006824 | α-amyrin | 426.8 | 7.35 | 1 | 1 | 39.51 | 1.37 | 1.2 | 0.76 | 0.23 | 3.06 | RRSM |
| MOL007036 | 5,6-dihydroxy-7-isopropyl-1,1-dimethyl-2,3-dihydrophenanthren-4-one | 298.41 | 4.38 | 2 | 3 | 33.77 | 1.19 | 0.8 | 0.29 | 0.29 | 14.91 | RRSM |
| MOL007041 | 2-isopropyl-8-methylphenanthrene-3,4-dione | 264.34 | 4.16 | 0 | 2 | 40.86 | 1.23 | 0.81 | 0.23 | 0.43 | 14.89 | RRSM |
| MOL007045 | 3α-hydroxytanshinoneⅡa | 310.37 | 3.56 | 1 | 4 | 44.93 | 0.53 | 0.22 | 0.44 | 0.3 | 23.78 | RRSM |
| MOL007048 | (E)-3-[2-(3,4-dihydroxyphenyl)-7-hydroxy-benzofuran-4-yl]acrylic acid | 312.29 | 3.21 | 4 | 6 | 48.24 | 0.18 | -0.89 | 0.31 | 0.4 | 8.87 | RRSM |
| MOL007049 | 4-methylenemiltirone | 266.36 | 4.33 | 0 | 2 | 34.35 | 1.25 | 0.87 | 0.23 | 0.38 | 14.6 | RRSM |
| MOL007050 | 2-(4-hydroxy-3-methoxyphenyl)-5-(3-hydroxypropyl)-7-methoxy-3-benzofurancarboxaldehyde | 356.4 | 3.58 | 2 | 6 | 62.78 | 0.35 | -0.73 | 0.4 | 0.24 | 7.89 | RRSM |
| MOL007051 | 6-o-syringyl-8-o-acetyl shanzhiside methyl ester | 628.64 | -1.13 | 5 | 16 | 46.69 | -1.73 | -2.08 | 0.71 | 0.22 | 9.94 | RRSM |
| MOL007058 | formyltanshinone | 290.28 | 3.36 | 0 | 4 | 73.44 | 0.54 | -0.28 | 0.42 | 0.41 | 24.12 | RRSM |
| MOL007059 | 3-beta-Hydroxymethyllenetanshiquinone | 294.32 | 3.16 | 1 | 4 | 32.16 | 0.38 | -0.48 | 0.41 | 0.36 | 22.51 | RRSM |
| MOL007061 | Methylenetanshinquinone | 278.32 | 4.26 | 0 | 3 | 37.07 | 1.03 | 0.46 | 0.36 | 0.36 | 24.33 | RRSM |
| MOL007063 | przewalskin a | 398.49 | 2.25 | 1 | 6 | 37.11 | -0.26 | -0.69 | 0.65 | 0.38 | 1.63 | RRSM |
| MOL007064 | przewalskin b | 330.46 | 3.18 | 1 | 4 | 110.32 | 0.34 | 0.22 | 0.44 | 0.32 | 2.17 | RRSM |
| MOL007068 | Przewaquinone B | 292.3 | 2.99 | 1 | 4 | 62.24 | 0.39 | -0.45 | 0.41 | 0.38 | 24.94 | RRSM |
| MOL007069 | przewaquinone c | 296.34 | 3.31 | 1 | 4 | 55.74 | 0.42 | -0.3 | 0.4 | 0.32 | 23.7 | RRSM |
| MOL007070 | (6S,7R)-6,7-dihydroxy-1,6-dimethyl-8,9-dihydro-7H-naphtho[8,7-g]benzofuran-10,11-dione | 312.34 | 2.34 | 2 | 5 | 41.31 | -0.06 | -0.68 | 0.45 | 0.32 | 22.54 | RRSM |
| MOL007071 | przewaquinone f | 312.34 | 2.07 | 2 | 5 | 40.31 | -0.09 | -0.9 | 0.46 | 0.29 | 22.45 | RRSM |
| MOL007077 | sclareol | 308.56 | 4.27 | 2 | 2 | 43.67 | 0.84 | 0.51 | 0.21 | 0.27 | 4.71 | RRSM |
| MOL007079 | tanshinaldehyde | 308.35 | 3.83 | 0 | 4 | 52.47 | 0.57 | -0.07 | 0.45 | 0.32 | 23.49 | RRSM |
| MOL007081 | Danshenol B | 354.48 | 2.59 | 1 | 4 | 57.95 | 0.53 | 0.11 | 0.56 | 0.3 | 4.28 | RRSM |
| MOL007082 | Danshenol A | 336.41 | 2.01 | 1 | 4 | 56.97 | 0.33 | -0.01 | 0.52 | 0.34 | 5.15 | RRSM |
| MOL007085 | Salvilenone | 292.4 | 4.26 | 0 | 2 | 30.38 | 1.46 | 1.07 | 0.38 | 0.35 | 20.81 | RRSM |
| MOL007088 | cryptotanshinone | 296.39 | 3.44 | 0 | 3 | 52.34 | 0.95 | 0.51 | 0.4 | 0.29 | 17.3 | RRSM |
| MOL007093 | dan-shexinkum d | 336.41 | 2.83 | 1 | 4 | 38.88 | 0.67 | -0.15 | 0.55 | 0.35 | 30 | RRSM |
| MOL007094 | danshenspiroketallactone | 282.36 | 3.24 | 0 | 3 | 50.43 | 0.88 | 0.51 | 0.31 | 0.34 | 15.19 | RRSM |
| MOL007098 | deoxyneocryptotanshinone | 298.41 | 4.32 | 1 | 3 | 49.4 | 0.85 | 0.24 | 0.29 | 0.3 | 27.17 | RRSM |
| MOL007100 | dihydrotanshinlactone | 266.31 | 2.77 | 0 | 3 | 38.68 | 1.26 | 0.81 | 0.32 | 0.38 | 5.42 | RRSM |
| MOL007101 | dihydrotanshinoneⅠ | 278.32 | 2.86 | 0 | 3 | 45.04 | 0.95 | 0.43 | 0.36 | 0.4 | 18.32 | RRSM |
| MOL007105 | epidanshenspiroketallactone | 284.38 | 2.37 | 0 | 3 | 68.27 | 0.9 | 0.61 | 0.31 | 0.33 | 1.77 | RRSM |
| MOL007107 | C09092 | 286.5 | 5.98 | 1 | 1 | 36.07 | 1.63 | 1.54 | 0.25 | 0.25 | -0.16 | RRSM |
| MOL007108 | isocryptotanshi-none | 296.39 | 3.59 | 0 | 3 | 54.98 | 0.93 | 0.34 | 0.39 | 0.3 | 31.92 | RRSM |
| MOL007111 | Isotanshinone II | 294.37 | 4.66 | 0 | 3 | 49.92 | 1.03 | 0.45 | 0.4 | 0.3 | 24.73 | RRSM |
| MOL007115 | manool | 304.57 | 5.5 | 1 | 1 | 45.04 | 1.28 | 1.16 | 0.2 | 0.28 | 5.81 | RRSM |
| MOL007118 | microstegiol | 298.46 | 4.75 | 1 | 2 | 39.61 | 1.05 | 0.99 | 0.28 | 0.33 | 4.52 | RRSM |
| MOL007119 | miltionone Ⅰ | 312.39 | 3.33 | 1 | 4 | 49.68 | 0.35 | -0.11 | 0.32 | 0.35 | 41.49 | RRSM |
| MOL007120 | miltionone Ⅱ | 312.39 | 2.14 | 1 | 4 | 71.03 | 0.62 | 0.03 | 0.44 | 0.28 | 2.91 | RRSM |
| MOL007121 | miltipolone | 300.43 | 2.74 | 1 | 3 | 36.56 | 0.5 | 0.17 | 0.37 | 0.3 | 1.7 | RRSM |
| MOL007122 | Miltirone | 282.41 | 4.73 | 0 | 2 | 38.76 | 1.23 | 0.87 | 0.25 | 0.32 | 14.82 | RRSM |
| MOL007123 | miltirone Ⅱ | 272.32 | 0.77 | 1 | 4 | 44.95 | 0.04 | -0.25 | 0.24 | 0.35 | 2.24 | RRSM |
| MOL007124 | neocryptotanshinone ii | 270.35 | 3.61 | 1 | 3 | 39.46 | 0.76 | 0.16 | 0.23 | 0.32 | 26.98 | RRSM |
| MOL007125 | neocryptotanshinone | 314.41 | 3.01 | 2 | 4 | 52.49 | 0.35 | -0.13 | 0.32 | 0.28 | 14.46 | RRSM |
| MOL007127 | 1-methyl-8,9-dihydro-7H-naphtho[5,6-g]benzofuran-6,10,11-trione | 280.29 | 3.21 | 0 | 4 | 34.72 | 0.5 | -0.27 | 0.37 | 0.33 | 37.89 | RRSM |
| MOL007130 | prolithospermic acid | 314.31 | 2.77 | 4 | 6 | 64.37 | 0.1 | -0.75 | 0.31 | 0.42 | 8.82 | RRSM |
| MOL007132 | (2R)-3-(3,4-dihydroxyphenyl)-2-[(Z)-3-(3,4-dihydroxyphenyl)acryloyl]oxy-propionic acid | 360.34 | 2.69 | 5 | 8 | 109.38 | -0.33 | -1.02 | 0.35 | 0.41 | 2.01 | RRSM |
| MOL007140 | (Z)-3-[2-[(E)-2-(3,4-dihydroxyphenyl)vinyl]-3,4-dihydroxy-phenyl]acrylic acid | 314.31 | 2.82 | 5 | 6 | 88.54 | -0.09 | -0.77 | 0.26 | 0.43 | 4.31 | RRSM |
| MOL007141 | salvianolic acid g | 340.3 | 2.2 | 4 | 7 | 45.56 | -0.14 | -0.97 | 0.61 | 0.45 | 2.4 | RRSM |
| MOL007142 | salvianolic acid j | 538.49 | 3.78 | 6 | 12 | 43.38 | -0.82 | -2.14 | 0.72 | 0.44 | 5.77 | RRSM |
| MOL007143 | salvilenone Ⅰ | 270.4 | 2.88 | 1 | 2 | 32.43 | 1.13 | 0.77 | 0.23 | 0.3 | 1 | RRSM |
| MOL007145 | salviolone | 268.38 | 4.05 | 1 | 2 | 31.72 | 1.04 | 0.72 | 0.24 | 0.36 | 0.33 | RRSM |
| MOL007149 | NSC 122421 | 300.48 | 4.99 | 1 | 2 | 34.49 | 1.08 | 0.63 | 0.28 | 0.29 | 14.56 | RRSM |
| MOL007150 | (6S)-6-hydroxy-1-methyl-6-methylol-8,9-dihydro-7H-naphtho[8,7-g]benzofuran-10,11-quinone | 312.34 | 2.42 | 2 | 5 | 75.39 | 0.03 | -0.74 | 0.46 | 0.29 | 23.45 | RRSM |
| MOL007151 | Tanshindiol B | 312.34 | 2.34 | 2 | 5 | 42.67 | 0.05 | -0.63 | 0.45 | 0.33 | 22.25 | RRSM |
| MOL007152 | Przewaquinone E | 312.34 | 2.34 | 2 | 5 | 42.85 | -0.04 | -0.65 | 0.45 | 0.32 | 22.44 | RRSM |
| MOL007154 | tanshinone iia | 294.37 | 4.66 | 0 | 3 | 49.89 | 1.05 | 0.7 | 0.4 | 0.31 | 23.56 | RRSM |
| MOL007155 | (6S)-6-(hydroxymethyl)-1,6-dimethyl-8,9-dihydro-7H-naphtho[8,7-g]benzofuran-10,11-dione | 310.37 | 3.57 | 1 | 4 | 65.26 | 0.44 | -0.31 | 0.45 | 0.29 | 23.48 | RRSM |
| MOL007156 | tanshinone Ⅵ | 296.34 | 2.44 | 2 | 4 | 45.64 | 0.48 | -0.28 | 0.3 | 0.38 | 15.21 | RRSM |
| MOL007134 | danshensu | 198.19 | 0.71 | 4 | 5 | 36.91 | -0.27 | -0.62 | 0.06 | 0.37 | 0.16 | RRSM |
| MOL000033 | (3S,8S,9S,10R,13R,14S,17R)-10,13-dimethyl-17-[(2R,5S)-5-propan-2-yloctan-2-yl]-2,3,4,7,8,9,11,12,14,15,16,17-dodecahydro-1H-cyclopenta[a]phenanthren-3-ol | 428.82 | 8.54 | 1 | 1 | 36.23 | 1.45 | 1.09 | 0.78 | 0 | 5.22 | RAPM |
| MOL000098 | quercetin | 302.25 | 1.5 | 5 | 7 | 46.43 | 0.05 | -0.77 | 0.28 | 0.38 | 14.4 | RAPM |
| MOL000211 | Mairin | 456.78 | 6.52 | 2 | 3 | 55.38 | 0.73 | 0.22 | 0.78 | 0.26 | 8.87 | RAPM |
| MOL000239 | Jaranol | 314.31 | 2.09 | 2 | 6 | 50.83 | 0.61 | -0.22 | 0.29 | 0.29 | 15.5 | RAPM |
| MOL000296 | hederagenin | 414.79 | 8.08 | 1 | 1 | 36.91 | 1.32 | 0.96 | 0.75 | 0 | 5.35 | RAPM |
| MOL000354 | isorhamnetin | 316.28 | 1.76 | 4 | 7 | 49.6 | 0.31 | -0.54 | 0.31 | 0.32 | 14.34 | RAPM |
| MOL000371 | 3,9-di-O-methylnissolin | 314.36 | 2.89 | 0 | 5 | 53.74 | 1.18 | 0.63 | 0.48 | 0 | 9 | RAPM |
| MOL000374 | 5'-hydroxyiso-muronulatol-2',5'-di-O-glucoside | 642.67 | -0.95 | 9 | 16 | 41.72 | -2.47 | -3.62 | 0.69 | 0 | 2.52 | RAPM |
| MOL000378 | 7-O-methylisomucronulatol | 316.38 | 3.38 | 1 | 5 | 74.69 | 1.08 | 0.84 | 0.3 | 0 | 2.98 | RAPM |
| MOL000379 | 9,10-dimethoxypterocarpan-3-O-β-D-glucoside | 462.49 | 0.74 | 4 | 10 | 36.74 | -0.63 | -1.5 | 0.92 | 0 | 13.06 | RAPM |
| MOL000380 | (6aR,11aR)-9,10-dimethoxy-6a,11a-dihydro-6H-benzofurano[3,2-c]chromen-3-ol | 300.33 | 2.64 | 1 | 5 | 64.26 | 0.93 | 0.55 | 0.42 | 0 | 8.49 | RAPM |
| MOL000387 | Bifendate | 418.38 | 2.56 | 0 | 10 | 31.1 | 0.15 | -0.06 | 0.67 | 0 | 17.96 | RAPM |
| MOL000392 | formononetin | 268.28 | 2.58 | 1 | 4 | 69.67 | 0.78 | 0.02 | 0.21 | 0 | 17.04 | RAPM |
| MOL000398 | isoflavanone | 316.33 | 2.42 | 2 | 6 | 109.99 | 0.53 | 0.17 | 0.3 | 0 | 15.51 | RAPM |
| MOL000417 | Calycosin | 284.28 | 2.32 | 2 | 5 | 47.75 | 0.52 | -0.43 | 0.24 | 0 | 17.1 | RAPM |
| MOL000422 | kaempferol | 286.25 | 1.77 | 4 | 6 | 41.88 | 0.26 | -0.55 | 0.24 | 0 | 14.74 | RAPM |
| MOL000433 | FA | 441.45 | 0.01 | 7 | 13 | 68.96 | -1.5 | -2.59 | 0.71 | 0 | 24.81 | RAPM |
| MOL000438 | (3R)-3-(2-hydroxy-3,4-dimethoxyphenyl)chroman-7-ol | 302.35 | 3.13 | 2 | 5 | 67.67 | 0.96 | 0.34 | 0.26 | 0 | 2.9 | RAPM |
| MOL000439 | isomucronulatol-7,2'-di-O-glucosiole | 626.67 | -0.68 | 8 | 15 | 49.28 | -2.22 | -3.36 | 0.62 | 0 | 0.93 | RAPM |
| MOL000442 | 1,7-Dihydroxy-3,9-dimethoxy pterocarpene | 314.31 | 3.11 | 2 | 6 | 39.05 | 0.89 | -0.04 | 0.48 | 0 | 7.95 | RAPM |
| MOL000407 | astragalosideⅣ | 785.09 | -0.35 | 9 | 14 | 22.5 | -2.11 | -3.41 | 0.15 | 0 |  | RAPM |
| MOL000310 | Denudatin B | 356.45 | 2.8 | 0 | 5 | 61.47 | 0.9 | 0.35 | 0.38 | 0.24 | 7.71 | RD |
| MOL000322 | Kadsurenone | 356.45 | 2.8 | 0 | 5 | 54.72 | 0.82 | 0.52 | 0.38 | 0.24 | 9.16 | RD |
| MOL000546 | diosgenin | 414.69 | 4.63 | 1 | 3 | 80.88 | 0.82 | 0.27 | 0.81 | 0.19 | 4.14 | RD |
| MOL000953 | CLR | 386.73 | 7.38 | 1 | 1 | 37.87 | 1.43 | 1.13 | 0.68 | 0.2 | 4.52 | RD |
| MOL001559 | piperlonguminine | 273.36 | 2.93 | 1 | 4 | 30.71 | 0.95 | 0.27 | 0.18 | 0.35 | 8.66 | RD |
| MOL001736 | (-)-taxifolin | 304.27 | 1.49 | 5 | 7 | 60.51 | -0.24 | -1.02 | 0.27 | 0.41 | 14.37 | RD |
| MOL005429 | hancinol | 372.5 | 2.46 | 1 | 5 | 64.01 | 0.53 | 0.17 | 0.37 | 0.25 | 4.06 | RD |
| MOL005430 | hancinone C | 400.51 | 3.34 | 0 | 6 | 59.05 | 0.74 | 0.06 | 0.39 | 0.19 | 4.14 | RD |
| MOL005435 | 24-Methylcholest-5-enyl-3belta-O-glucopyranoside_qt | 400.76 | 7.63 | 1 | 1 | 37.58 | 1.33 | 0.96 | 0.72 | 0.22 | 4.91 | RD |
| MOL005438 | campesterol | 400.76 | 7.63 | 1 | 1 | 37.58 | 1.34 | 0.95 | 0.71 | 0.22 | 4.83 | RD |
| MOL005440 | Isofucosterol | 412.77 | 7.83 | 1 | 1 | 43.78 | 1.36 | 0.97 | 0.76 | 0.24 | 5.18 | RD |
| MOL005458 | Dioscoreside C_qt | 444.72 | 3.96 | 2 | 4 | 36.38 | 0.39 | -0.44 | 0.87 | 0.19 | 5.49 | RD |
| MOL005461 | Doradexanthin | 584.96 | 8.73 | 2 | 3 | 38.16 | 0.52 | -1.19 | 0.54 | 0.34 | 4.13 | RD |
| MOL005463 | Methylcimicifugoside_qt | 556.81 | 3.21 | 1 | 7 | 31.69 | 0.21 | -0.41 | 0.24 | 0.23 | 11.29 | RD |
| MOL005465 | AIDS180907 | 394.45 | 4.81 | 3 | 6 | 45.33 | 0.73 | 0 | 0.77 | 0.27 | 14.86 | RD |
| MOL001494 | Mandenol | 308.56 | 6.99 | 0 | 2 | 42 | 1.46 | 1.14 | 0.19 | 0.25 | 5.39 | FC |
| MOL001495 | Ethyl linolenate | 306.54 | 6.55 | 0 | 2 | 46.1 | 1.54 | 1.12 | 0.2 | 0.25 | 6.2 | FC |
| MOL002883 | Ethyl oleate (NF) | 310.58 | 7.44 | 0 | 2 | 32.4 | 1.4 | 1.1 | 0.19 | 0.19 | 4.85 | FC |
| MOL003137 | Leucanthoside | 462.44 | -0.07 | 7 | 11 | 32.12 | -1.27 | -2.41 | 0.78 | 0 | 16.28 | FC |
| MOL005481 | 2,6,10,14,18-pentamethylicosa-2,6,10,14,18-pentaene | 342.67 | 9.51 | 0 | 0 | 33.4 | 1.94 | 1.99 | 0.24 | 0.29 | 6.05 | FC |
| MOL005486 | 3,4-Dehydrolycopen-16-al | 548.92 | 11.48 | 0 | 1 | 46.64 | 2 | 0.6 | 0.49 | 0.37 | 4.29 | FC |
| MOL005489 | 3,6-Digalloylglucose | 484.4 | -0.03 | 9 | 14 | 31.42 | -1.95 | -3.05 | 0.66 | 0.38 | 3.39 | FC |
| MOL005503 | Cornudentanone | 378.56 | 4.97 | 0 | 5 | 39.66 | 0.47 | 0.09 | 0.33 | 0.21 | 2.83 | FC |
| MOL005530 | Hydroxygenkwanin | 300.28 | 2.32 | 3 | 6 | 36.47 | 0.52 | -0.44 | 0.27 | 0.31 | 15.22 | FC |
| MOL005531 | Telocinobufagin | 402.58 | 2.11 | 3 | 5 | 69.99 | -0.12 | -0.85 | 0.79 | 0.26 | 5.15 | FC |
| MOL008457 | Tetrahydroalstonine | 352.47 | 2.66 | 1 | 4 | 32.42 | 0.9 | 0.33 | 0.81 | 0.23 | 10.55 | FC |
| MOL005552 | gemin D | 634.49 | 0.73 | 11 | 18 | 68.83 | -2.17 | -2.71 | 0.56 | 0.38 | 5.55 | FC |
| MOL005557 | lanosta-8,24-dien-3-ol,3-acetate | 468.84 | 8.5 | 0 | 2 | 44.3 | 1.45 | 1.31 | 0.82 | 0.25 | 7.21 | FC |
| MOL005546 | cornuside | 542.54 | -0.71 | 7 | 14 | 2.61 | -2.09 | -2.98 | 0.71 | 0.3 |  | FC |
| MOL001680 | Loganin | 390.43 | -2.08 | 5 | 10 | 5.9 | -1.48 | -2.26 | 0.44 | 0.24 |  | FC |
| MOL002819 | catalpol | 362.37 | -3.77 | 6 | 10 | 5.07 | -1.72 | -2.33 | 0.44 | 0.22 |  | RR |
| MOL004355 | Spinasterol | 412.77 | 7.64 | 1 | 1 | 42.98 | 1.44 | 1.04 | 0.76 | 0.21 | 5.32 | RT |
| MOL006756 | Schottenol | 414.79 | 8.08 | 1 | 1 | 37.42 | 1.33 | 0.91 | 0.75 | 0.22 | 5.63 | RT |
| MOL000422 | kaempferol | 286.25 | 1.77 | 4 | 6 | 41.88 | 0.26 | -0.55 | 0.24 | 0 | 14.74 | RRG |
| MOL000787 | Fumarine | 353.4 | 2.95 | 0 | 6 | 59.26 | 0.56 | -0.13 | 0.83 | 0.3 | 23.46 | RRG |
| MOL003648 | Inermin | 284.28 | 2.44 | 1 | 5 | 65.83 | 0.91 | 0.36 | 0.54 | 0.3 | 11.73 | RRG |
| MOL004492 | Chrysanthemaxanthin | 584.96 | 8.24 | 2 | 3 | 38.72 | 0.51 | -0.98 | 0.58 | 0.3 | 17.47 | RRG |
| MOL005308 | Aposiopolamine | 271.34 | 1.39 | 1 | 4 | 66.65 | 0.66 | 0.4 | 0.22 | 0.35 | 3.54 | RRG |
| MOL005314 | Celabenzine | 379.55 | 2.29 | 2 | 5 | 101.88 | 0.77 | 0.05 | 0.49 | 0.35 | 8.15 | RRG |
| MOL005317 | Deoxyharringtonine | 515.66 | 3.13 | 1 | 9 | 39.27 | 0.19 | -0.25 | 0.81 | 0.23 | 7.9 | RRG |
| MOL005318 | Dianthramine | 289.26 | 2.05 | 5 | 7 | 40.45 | -0.23 | -0.97 | 0.2 | 0.42 | 5.14 | RRG |
| MOL005320 | arachidonate | 304.52 | 6.41 | 1 | 2 | 45.57 | 1.27 | 0.58 | 0.2 | 0.26 | 7.56 | RRG |
| MOL005321 | Frutinone A | 264.24 | 2.7 | 0 | 4 | 65.9 | 0.89 | 0.46 | 0.34 | 0.47 | 19.1 | RRG |
| MOL005344 | ginsenoside rh2 | 622.98 | 4.04 | 6 | 8 | 36.32 | -0.51 | -1.38 | 0.56 | 0.24 | 11.08 | RRG |
| MOL005348 | Ginsenoside-Rh4_qt | 458.8 | 5.59 | 3 | 3 | 31.11 | 0.5 | -0.18 | 0.78 | 0.25 | 6.97 | RRG |
| MOL005356 | Girinimbin | 263.36 | 4.6 | 1 | 1 | 61.22 | 1.72 | 1.22 | 0.31 | 0.33 | 8.17 | RRG |
| MOL005357 | Gomisin B | 514.62 | 2.73 | 1 | 9 | 31.99 | 0.6 | 0.18 | 0.83 | 0.19 | 7.81 | RRG |
| MOL005376 | Panaxadiol | 460.82 | 5.46 | 2 | 3 | 33.09 | 0.82 | 0.23 | 0.79 | 0.22 | 6.34 | RRG |
| MOL005384 | suchilactone | 368.41 | 3.73 | 0 | 6 | 57.52 | 0.82 | 0.28 | 0.56 | 0.28 | 9.03 | RRG |
| MOL005399 | alexandrin_qt | 414.79 | 8.08 | 1 | 1 | 36.91 | 1.3 | 0.88 | 0.75 | 0.23 | 5.53 | RRG |
| MOL005401 | ginsenoside Rg5_qt | 442.8 | 6.8 | 2 | 2 | 39.56 | 0.88 | 0.21 | 0.79 | 0.24 | 5.65 | RRG |
| MOL005361 | Malonylginsenoside Rc | 1165.48 | -0.52 | 14 | 25 | 7.84 | -4.27 | -5.65 | 0.03 | 0.25 |  | RRG |
